# The Comparison between Different Extracellular Vesicle Isolation Methods by AFM-IR Nanospectroscopy

**DOI:** 10.1021/acs.analchem.5c07289

**Published:** 2026-03-10

**Authors:** Jéssica Verônica da Silva, Otávio Berenguel, Raquel Silva Neres-Santos, Herculano da Silva Martinho, Marcela Sorelli Carneiro-Ramos

**Affiliations:** † Centro de Ciências Naturais e Humanas (CCNH), 74362 Universidade Federal do ABC, Av. dos Estados 5001, Santo André, SP 09210-580, Brazil; ‡ Laboratório Nacional de Nanotecnologia (LNNano), Centro Nacional de Pesquisa em Energia e Materiais (CNPEM), R. Giuseppe Máximo Scolfaro 10000, Campinas, SP 13083-100, Brazil

## Abstract

Extracellular vesicles (EVs) contain cell-type-specific signatures and have been proposed as biomarkers in various diseases. However, due to their small dimensions, quantification and size distribution, the biophysical characterization of these particles is challenging and still controversial. The diffraction-limiting effect limits acquiring spectral data on EV samples in the mid-IR range, making it impossible to capture images and spectra at the nanoscale by conventional vibrational spectroscopy methods. Here, we employed atomic force microscopy–infrared nanospectroscopy (AFM-IR) technique to elucidate the molecular signatures of EVs isolated from blood (serum and plasma), as well as the direct correlation of their topography and biochemical signature with respect to the isolation method. EVs were extracted from serum or plasma from C57BL/6 male mice by Total Exosome Isolation Reagent (TEIR), ultracentrifugation, and size-exclusion chromatography (SEC), and evaluated by microreflectance in FTIR and AFM-IR analysis. The multivariate analysis by PCA and PLS-DA showed that phosphate groups could be significantly altered depending on the EV isolation/purification method, as well as glycosidic linkages. The SEC processing strongly depletes the amount of biomolecules whose bands fall in the region above 1150 cm^–1^, which includes α-helix and fatty acid content of EVs, while the β-sheet and random coil/turn/loop conformational content of proteins, nucleic acids, phospholipids, carbohydrates, and glycoproteins is almost preserved for SEC. The TEIR method can sustain the diverse fractions of conformational content of proteins at the cost of damaging nucleic acids, phospholipids, carbohydrates, and glycoproteins. Still, the UC processing was able to preserve the largest amount of biomolecules present in EVs. However, structural damage on membranes was observed, e.g., smearing out of phospholipids related to membrane-packing bands (1720–1800 cm^–1^ region) and an overall decrease in protein bands decreasing.

## Graphical abstract

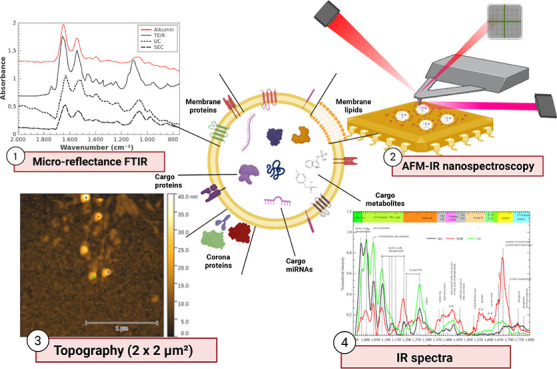

## Introduction

Extracellular vesicles (EVs) are lipid bilayer, membrane-delimited, nano- to microsized structures that appear to be released by all kinds of cells.^1^ The current literature on EVs reports that they can be isolated from culture cell medium, blood (plasma or serum*),* urine, milk, saliva, embryos, and microgreens.^2,3^ EVs contain cell-type-specific signatures and have been proposed as biomarkers in a variety of diseases.^4,35^ In this sense, blood-based EVs can be used as minimally invasive diagnostic, prognostic, and predictive tools.^4^

Since the 90s, technological advances, such as nanoparticle tracking analysis (NTA), dynamic light scattering (DLS), and high-resolution flow cytometry, have increased the research output in EV investigations.^3^ Also, biochemical composition analysis and microscopy techniques have led to a deeper understanding of EVs.^1^

On the other hand, the use of specific and isolated markers in canonical biomolecular techniques (such as Western blotting or flow cytometry) carries risks related to EV population investigation, as it is complex to select an isolated marker that may accurately represent the isolated EVs, necessitating further characterization by orthogonal analysis using different markers for the same population.^3,4^ However, due to their small dimensions and size distribution, EV quantification and their biophysical characterization are complex and still controversial.^3,7,20^

EVs’ isolation process from plasma or serum is a challenge due to the inherent complexity of blood. The high abundance of albumin and other soluble blood proteins causes contamination in EV pellets.^5^ It is important to address that different purification methods are capable of delivering complex and sometimes conflicting outcomes, which directly impact the growth of knowledge about EVs.^3^ Also, it is well-known that different EVs’ isolation methodologies impact their spectral fingerprints.^1,2^

According to Ter-Ovanesyan et al.,^6^ there is no unique optimal way to isolate EVs, since the purification method must match the application. It is crucial to have effective ways of comparing both the yield and the purity of different isolation methods. Thus, it is important to develop techniques and apply them to detect and discriminate between changes in the composition of EVs obtained under different protocols and classify them according to the isolation method.^3^

Biospectroscopy methods based on vibrational spectroscopy (such as infrared absorption) are widely used options for *in situ* characterization of biosamples due to their molecular and structural sensitivity.^8,9^ However, light diffraction effects limit imaging of submicron samples, especially in the mid-IR range, which makes it impossible to capture images and spectra at the nanoscale by conventional spectrometers such as Fourier transform infrared spectroscopy (FTIR).^10^

Nanoscale infrared spectroscopy (IR) is an advanced analytical technique that combines infrared spectroscopy with high-resolution microscopy to characterize materials at the nanometer scale.^11^ Traditional IR spectroscopy provides chemical information based on molecular vibrations but is limited by optical diffraction, restricting its spatial resolution to a few micrometers. Nanoscale IR techniques, such as scattering-type scanning near-field optical microscopy (s-SNOM), photothermal-induced resonance (PTIR), and photoinduced force microscopy (PiFM), overcome this limitation by using sharp atomic force microscopy (AFM) tips to enhance spatial resolution down to tens of nanometers. These methods enable the detailed investigation of nanoscale chemical composition, molecular interactions, and phase distributions in diverse materials, including polymers, biological samples, and nanostructures, making them invaluable for research in nanotechnology, materials science, and biomedicine.^3,11^

Therefore, the nanoscale IR using AFM (AFM-IR) has the potential to discriminate the molecular composition of EVs, making a direct correlation to their topography, applying mid-IR spectroscopy for the advancement of the understanding of EV structure, composition, and function.^2,3,10^ However, at the nanoscale, it is unclear how the different isolation methodologies could affect EV spectra due to the lack of a defined assignment of vibrational bands of EVs. Here, we present a detailed investigation about the role of the preparation process in the AFM-IR spectra of EVs isolated from plasma or serum samples from mice, aiming to identify intrinsic and extrinsic bands of EVs at the nanoscale.

## Materials and Methods

### Blood-Based Samples

The experiments were carried out following Brazilian Federal Law No. 6,638, of 1979, which regulates the use of animals in scientific experimentation, under the protocol of the Research Ethics Committee of the Federal University of ABC (3192040123). Male C57BL/6 mice were used, aged between 6 and 8 weeks, weighing between 20 and 28 g. All animals were placed in collective cages containing a maximum of five animals, with an artificial light/dark cycle of 12 h, at a constant room temperature of 25 °C, with abundant water and food supplements. The plasma samples were obtained from the total blood that was collected with 0.5 M EDTA by puncturing the inferior vena cava and differential centrifugation at 300, 3000, and 20,000*g* for 15 min. Serum samples were obtained from the blood, free of anticoagulant, and centrifuged at 12.000*g* for 10 min at 4 °C. Plasma and serum were stored at −80 °C until isolation.

Three samples per method were pooled, and the EVs were isolated using the following methods:

#### Total Exosome Isolation Reagent from Serum Samples

The isolation was carried out according to the Total Exosome Isolation Reagent (TEIR) manufacturer’s instructions.^22^ Briefly, the first step involves centrifugation of the serum sample at 2000*g* for 30 min to remove cells and debris. The supernatant containing the clarified serum was then transferred to a new tube without disturbing the pellet, where 20% (relative to the total volume of serum) of the TEIR reagent was added, mixed, and incubated for 30 min at 4 °C. After incubation, the sample was centrifuged at 10,000*g* for 10 min at room temperature. The pellet was resuspended in 1x filtered phosphate-buffered saline (PBS) which was exosome-free, and filtered using a membrane filter (PES 0.1 μm), and then diluted to 1:10 with ultrapure PES 0.1 μm-filtered water.

#### Ultracentrifugation Using Plasma Samples

Differential ultracentrifugation employs high g-force to separate the EVs with different sedimentation rates based on their size and density,^29^ being the gold standard to isolate and concentrate EVs.^30^ In the ultracentrifugation (UC) method, plasma samples were ultracentrifuged twice at 100,000*g* for 1 h and 30 min at 4 °C. Then, they were resuspended in 1 mL of 1X filtered PBS and washed by spinning a second time at 100,000*g* for 1 h and 30 min at 4 °C. The pellets were resuspended in 500 μL of 1X filtered PBS and then diluted to 1:10 with filtered ultrapure water.

#### Size-Exclusion Chromatography Using Plasma Samples

The isolation of EVs from plasma samples was done by size exclusion chromatography (SEC) using qEV1 70 nm Gen 2 columns (Izon). The fractions 7–10, enriched with small EVs, were chosen to proceed with concentration by UC. These fractions were pooled using a single ultracentrifugation at 100,000*g* for 1 h and 30 min at 4 °C. The pellets were resuspended in 50 μL of 1X filtered PBS and then diluted to 1:10 with filtered ultrapure water. The need to achieve a higher EV concentration and improve downstream results increased according to the sensitivity of EV analysis. In this sense, all techniques provide EV recovery yield and purity in different grades, where the principle of SEC is separation based on a difference in size, a “resin filtration”, and is also reported by the lower contamination scores reported in different analyses.^32–34^

### Albumin

In recent years, the study of blood proteins has increased due to their role in EVs uptake, as these proteins are more than just contaminants but also a part of the EVs corona.^12,13^ In this way, considering the albumin spectral contribution to EVs spectra is of fundamental relevance.

Mouse albumin from mouse serum (Sigma-Aldrich, A3139) was also prepared in a 1:1 ratio in 1X filtered PBS for spectral comparison.

### FTIR Measurements

EVs from the three isolation methods and the mouse albumin sample were measured and compared. Analysis was performed on 1 mL of drop-casted EVs that was dried for 10 min on Pt substrates. Measurements were performed using the FTIR microspectrometer (FT-IR 660 – Varian Inc.) using a 24 μm^2^ of area of the Ge detector, by microreflectance. The resolution was set to 4 cm^–1^, while the number of scans was set to 100.

### AFM-IR Measurements

EV samples were thawed at room temperature in a laminar flow to avoid contamination. 2 μL of EV solution was dropped onto a flat gold substrate; the topography images and infrared spectra (IR) were obtained using nanoIR2-s equipment from Bruker in contact mode. A BudgetSensors ContGB-G probe with a nominal spring constant of 0.2 N/m and <50.0 nm tip end radius was used for the scanning. The infrared source used in the measurements was a Daylight Solutions MIRcat-QT-2400 quantum cascade laser (QCL), operating in the spectral range 950–1920 cm^–1^. Each data point was acquired from 3 individual scans, with a total of 32 spectrum coaverages performed to enhance the signal-to-noise ratio. A total of 40 EVs were sampled for each extraction method.

## Data Analysis

### Principal Components Analysis (PCA)

The classical Principal Components Analysis (PCA)^23^ was performed on a mean-centered raw data of at least 30 particles per sample to extract outliers and identify potential experimental bias. All spectral analysis steps were performed in the R software using the *ChemSpec* vignette.^24^ The reduced Q-residual and T^2^ Hotelling’s statistics were used to identify outliers. Reduced Q-residuals measure the difference between a sample and its projection on the retained factors of the model. Examining reduced Q-residuals permits the detection of significant residual outliers. In contrast, Hotelling’s *T* ^2^ value measures the variation in each sample within the model, indicating how far each sample is from the model’s center (scores = 0). It is a measure of score outliers. Raw spectral data were examined using the *T* ^2^ Hotelling versus Q-residuals (reduced) graph.

### Partial Least Squares: Discriminant Analysis (PLS-DA)

After outlier removal, all remaining spectra were preprocessed to make them statistically comparable. Baseline correction was performed using a curve-fitting method proposed by Lieber and Mahadevan–Jansen,^25^ based on a least-squares polynomial curve fitting. All spectra were normalized and scaled using total intensity normalization.^26^ The PLS-DA analysis was then carried out. It is a multivariate supervised approach that predicts class membership using the linear regression of original data. We employed the *plsr* function from the R pls package^25,26^ to perform the PLS regression. The classification and cross-validation were performed using the caret package equivalent wrapper function.^27^ A permutation test was used to evaluate the performance of class discrimination. A PLS-DA model was constructed between the data and the permuted class labels in each permutation, using the optimal number of components determined via leave-one-out cross-validation for the model based on the original class assignment. The classification accuracy, *R* ^2^, and *Q* ^2^ were used to assess the performance of class discrimination. The first is determined by the prediction accuracy. The *R* ^2^ parameter measures how effectively the model fits accurate data based on the ratio between and within-group sums of squares. *Q*,^2^ on the other hand, is the “predictability” or projected variation obtained from cross-validation. Excellent forecasts will feature a high *Q*,^2^ whereas *Q* ^2^‘s negative indicates that the model is either not predictive or overfitted.^26–28^ In the PLS-DA model, two quantifiers were utilized to assess the relevance of the vibrational band frequency. The first is the variance importance projection (VIP) score, which is a weighted sum of squares of the PLS loadings that considers the amount of explained spectral intensity fluctuation in each dimension. The other measure of relevance is based on the weighted sum of the PLS regression. The weights are determined by dividing the sums of squares by the number of PLS components. The exact number of predictors will be constructed for each group in multiple-group analysis, and the average of the feature coefficients will be utilized to represent the overall coefficient-based relevance.

### Sample Size

Sample size adequacy was evaluated through resampling stability^38^ and permutation-based power analysis^39^ based on our spectral data. Classification performance stabilized around 30 samples per group. Empirical statistical power exceeded 80% for sample sizes above 30 per group, indicating that the study design is sufficiently powerful to detect multivariate group differences.

## Results and Discussion

### FTIR Spectroscopy

Figure 1 shows the FTIR spectra from EVs isolated by the three different methods, along with the mouse albumin spectra. The mouse albumin spectra are shown in Figure 1. Thery are dominated by intense Amide I (1650 cm^–1^) and Amide II (1550 cm^–1^) bands, followed by υPO_2_^–^ (1070 cm^–1^), which would be PBS buffer, CH_2_ and CH_3_ scissoring (1450 cm^–1^), and amino acids signatures bands, as expected.^14,15^ We noticed that all EV samples presented albumin bands. Bands related to biomolecules, such as nucleic acids, membrane lipids, and proteins, were observed in all EV samples. However, as already described,^18^ even if the formation of PBS crystal salts could be avoided using micro-dimension techniques, the isolation of EVs from other non-EV carriers, e.g., those containing blood proteins, cannot be achieved, and they will be detected in far-field IR spectroscopy. Therefore, the EV fingerprint itself may still be hidden in the spatially averaged IR spectra acquired on large sample areas.

**Figure 1:**
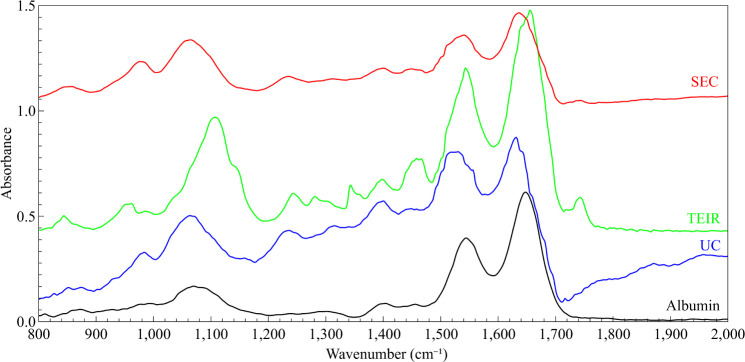
FTIR average spectra obtained from size-exclusion chromatography (SEC), Total Exosome Isolation Reagent (TEIR), and ultracentrifugation (UC) EV samples, and mouse serum albumin.

The TEIR EV ester group vibration at 1745 cm^–1^ is the most intense band. It is related to lipids, cholesterol, phospholipids, and oligosaccharides, which might interact with membranes, membrane-bound oligosaccharides, polysaccharides, and also DNA signatures, which could be derived from the isolation of EVs from other compounds.

Additionally, the EV isolation by UC directly impacts the intensity of the spectra, since the UC isolation process delivers fewer micrograms of EVs.^19^ Bands assigned to cholesterol, polypeptides, and RNA were present as well. The phosphate from PBS presents a signal overlap with the nucleic acid region in the 1230 cm^–1^ region. However, an overall red shift was observed in bands in the 1000–1700 cm^–1^ spectral window in comparison to albumin and TEIR spectra.

SEC-isolated EV FTIR spectra were very similar to the albumin one. Otherwise, in comparison with the UC method, the same red-shift band was observed, indicating that there is a chemical modification of TEIR in comparison to the UC and SEC samples. Bands related to Amide I and II, as seen in albumin spectra, as well as ester groups of triglycerides, cholesterol, lipids, polypeptides, phosphate/DNA/RNA/PBS, and RNA were observed. Also, bands related to phosphate and DNA, which are provided by the PBS buffer and the isolation method, respectively, were also detected.

As described by Di Santo et al.,^3^ a challenge in considering extracellular vesicles is their heterogeneity, resulting from their purification and characterization, but also from their biogenesis, even in *in vitro* studies.^20^ On the other hand, it is important to address that none of the EV purification methods fits all analyses, and it is important to follow reproducible steps. Di Santo et al.^3^ provided a rich schematic spectrum that corroborates what we present in this paper. However, it is also possible to see that the albumin signature is expected in *in vivo* and *in vitro* studies at the microscale. Another challenge, considering the microscale analysis, was the 1x PBS crystals formed after the drying of the sample, limiting the analysis to the samples’ edges (Figure 2a).

**Figure 2:**
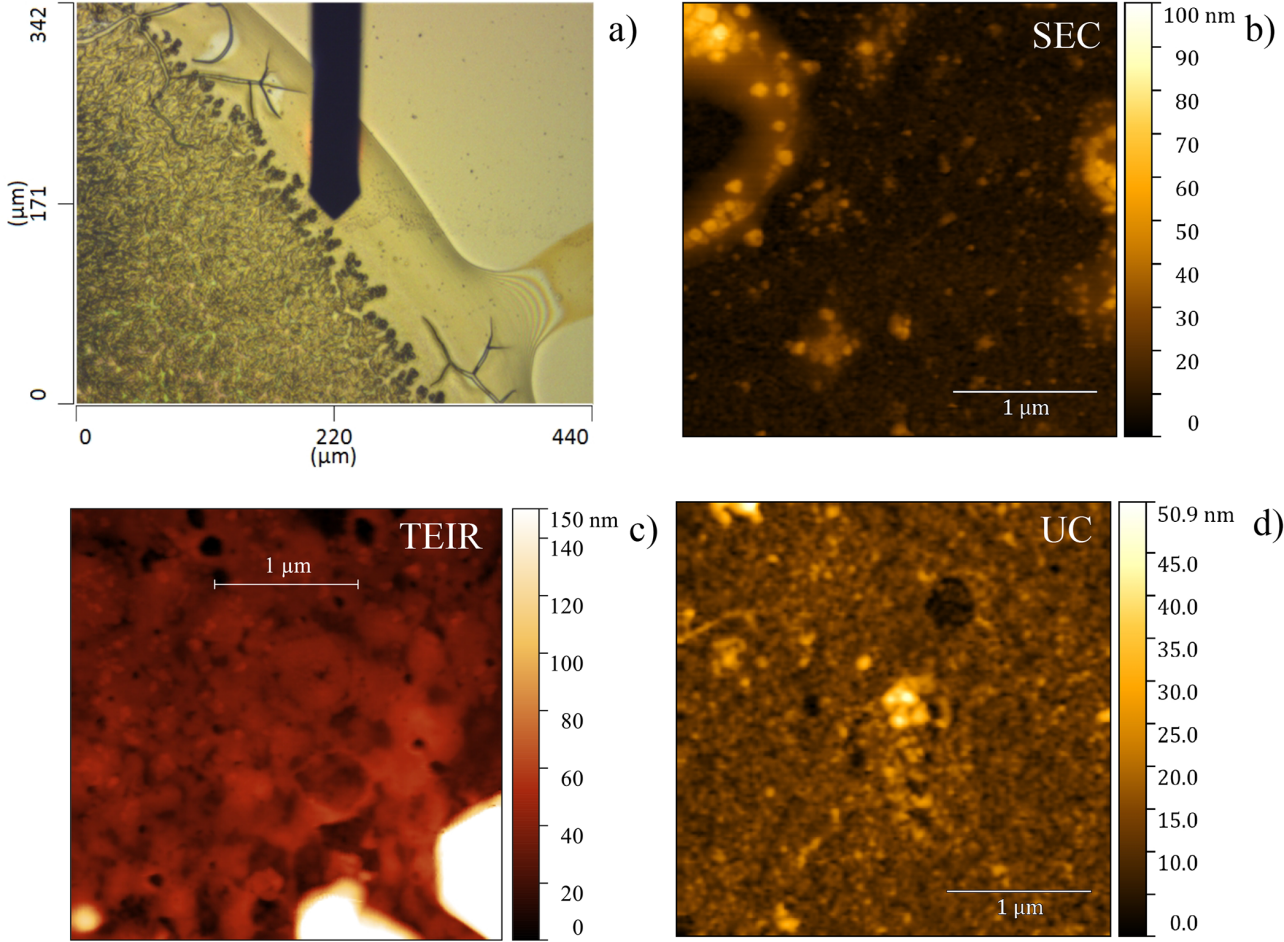
(a) Stereomicroscopy (40×) image from a single drop of an EV sample, where the PBS 1x buffer crystals are visible, along with the border of the drop, which is clearer than the center. (b–d) AFM-IR topographic images (5 × 5 μm^2^) acquired from SEC EVs (b), TEIR (c), and UC (d) samples.

### AFM-IR Measurement of EVs

The same set of samples was analyzed using AFM-IR, obtaining the topography image (Figures 2 and 3) and its infrared spectra in contact mode (Figure 3). The drying pattern of the sample diluted in 1x PBS (Figure 2a) shows crystalline formation of the salts in the drop, preventing AFM-IR measurements on this site. As shown in Figure 2b–d, there was no clear delineation of single EVs. The spectral noise from the albumin signature is still present in the infrared spectra at the nanoscale, indicating that even when avoiding macroaggregates of albumin, it is still at the EVs interface (Figure 3a–c).

**Figure 3:**
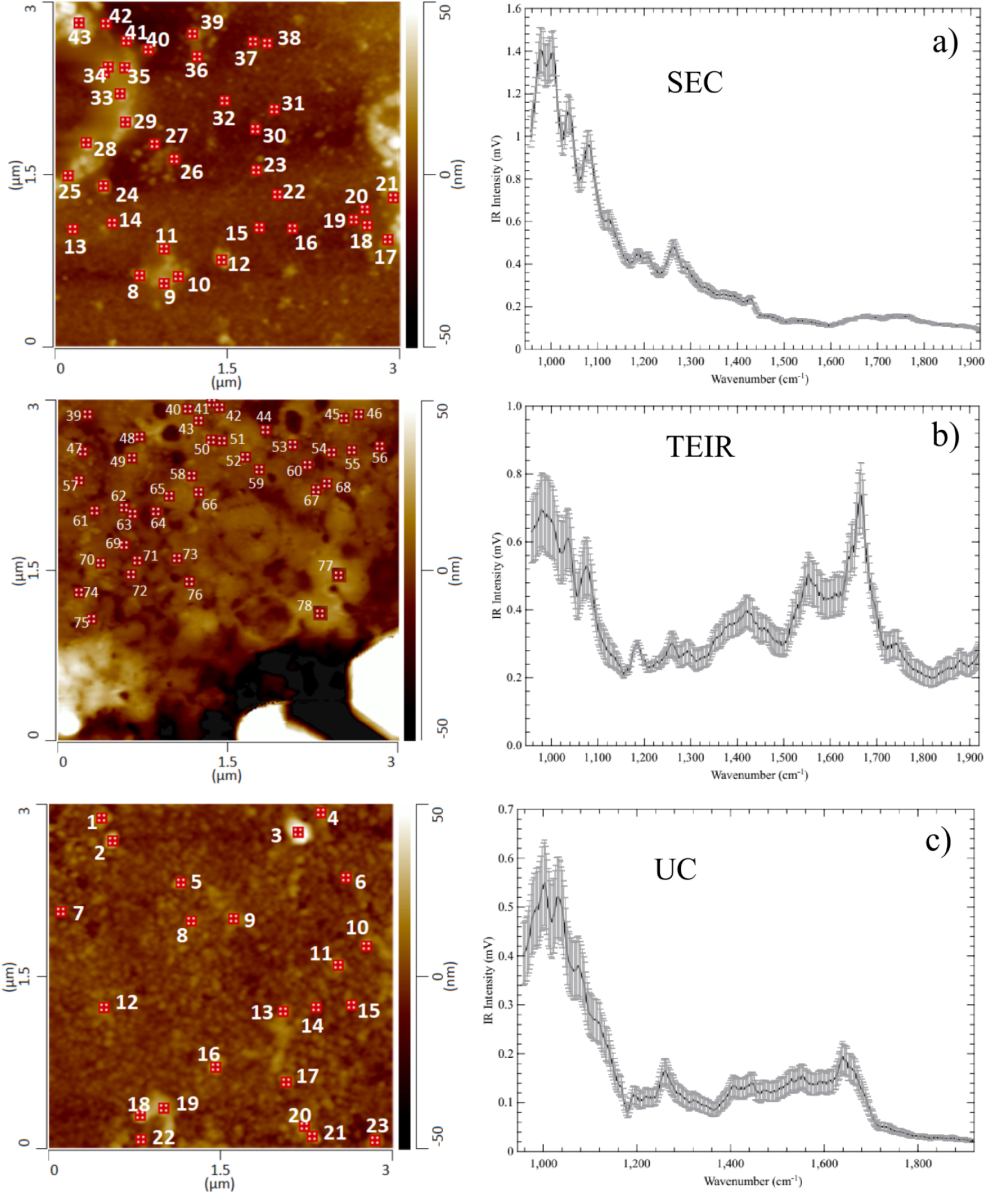
Box plot for AFM-IR spectra acquired in points indicated on the images to the left for SEC (a), TEIR (b), and UC (c) samples, diluted in ultrapure water (1:10). Spectra were acquired from a set of 50 EVs (only representative points are shown).

### Size Exclusion Chromatography (SEC)

As shown in the AFM-IR results (Figure 3a), SEC EVs isolated in 1:10 ultrapure water presented the topography of single particles and demonstrated great molecular homogeneity between the vesicles. It is interesting to notice the strong intensity depletion of the Amide II band, despite it being detectable in FTIR at the microscale (Figure 1) and in previous mid-IR range studies.^35^ This fact indicates a special concern about the SEC methodology due to the removal of highly abundant proteins from blood-based EVs.^29^

Lipids, fatty acid esters, methylene chains in lipids, and nucleic acids contribute to the specific vibration in SEC samples.

### Total Exosome Isolation Reagent from Serum

Bands related to lipids and nucleic acids, such as RNA, were observed in the TEIR sample, including protein bands Amide I and Amide II. The observed topography in this concentration highly correlates with the topography of albumin.^21^ Most single particles could contrast with the background substrate at a 1:10 dilution. Different sample regions were chosen to differentiate potentially isolated EVs from other bioaggregates as well. An intense band related to fatty acids remained at 1745 cm^–1^, and bands related to proteins such as Amides I and II were also observed, while nucleic acid bands could also be observed.

Even though the topography was better for isolated vesicles, it is visible how the surface of these vesicles continues to interact with blood proteins. In this way, as described by the TEIR User Guide,^22^ the precipitation is the principle of the isolation method, based on tying up water molecules, where the reagent forces less soluble components, such as exosomes, out of solution, allowing them to be collected after brief, low-speed centrifugation. On the other hand, without an additional purification method, this may also permit other hydrophilic serum compounds to precipitate.

### Ultracentrifugation Using Plasma Samples

In the UC samples, two different regions were chosen to collect topography and spectra, since there were fewer well-delineated vesicles (Figure 2c), as expected from the literature.^30,32^ Besides, this type of methodology has lower purity due to protein or EV aggregation, making it harder to further distinguish the EV subpopulation.^30^ According to microreflectance FTIR spectra, albumin has a great contribution to the overall spectra. However, according to AFM-IR results, there is strong heterogeneity among the UC EVs, even though intense bands related to proteins (1665 and 1520 cm^–1^) are conserved in most of them. On the other hand, heterogeneity within the same population of isolated EVs is expected even in *in vitro* studies using one type of cell lineage, as previously observed,^20^ since the EV signature will reflect their cargo.

Previous studies have been dedicated to describing the advantages and disadvantages of UC,^30,33^ with the conclusion that the lower purity, due to the aggregation of proteins during UC, impacts downstream analysis, making it preferable to combine two or more types of isolation methods, such as SEC, to obtain a greater amount of pure EVs.^31^ Our findings strongly corroborate this, highlighting the challenge of distinguishing isolated EVs from other plasma compounds.

### Multivariate Analysis

Data mining of the complete AFM-IR spectral set was performed. The PCA analysis results were summarized in Figure 4. We noticed that SEC, TEIR, and UC methods of EV separation present very distinctive spectral features, enabling a high degree of discrimination among spectra from these samples (Figure 4a). In particular, the PC1 vs. PC2 combination enabled >90% discrimination of groups. Biplot inspection (Figure 4b) shows that bands at 967–977 cm^–1^ (symmetric stretching mode of the phosphate ion) and 1025–1031 cm^–1^ (glycosidic linkages) present the greatest contribution to this discrimination. Clustering analysis shown in the dendrogram (Figure 4c) clearly indicated the formation of 3 clusters of data, mainly composed of SEC, TEIR, and UC samples. Phosphate groups are essential components of the cell membrane and could be significantly altered depending on the EV isolation/purification method. Critical is the role of glycosidic linkages. Glycosidic linkages are crucial in EVs as they form the basis of their glycans, which are involved in EV biogenesis, cell recognition, and uptake.^37^ These sugar-based bonds link monosaccharides together and also connect sugars to proteins (glycoproteins) or lipids (glycolipids) on the EV surface. The specific patterns of these linkages influence EV function, such as how they interact with recipient cells, and can be altered in disease, making them potential biomarkers or therapeutic targets. Changes in glycosidic linkages due to the EV isolation/purification method could mask relevant therapeutic routes based on EVs.^37^

**Figure 4:**
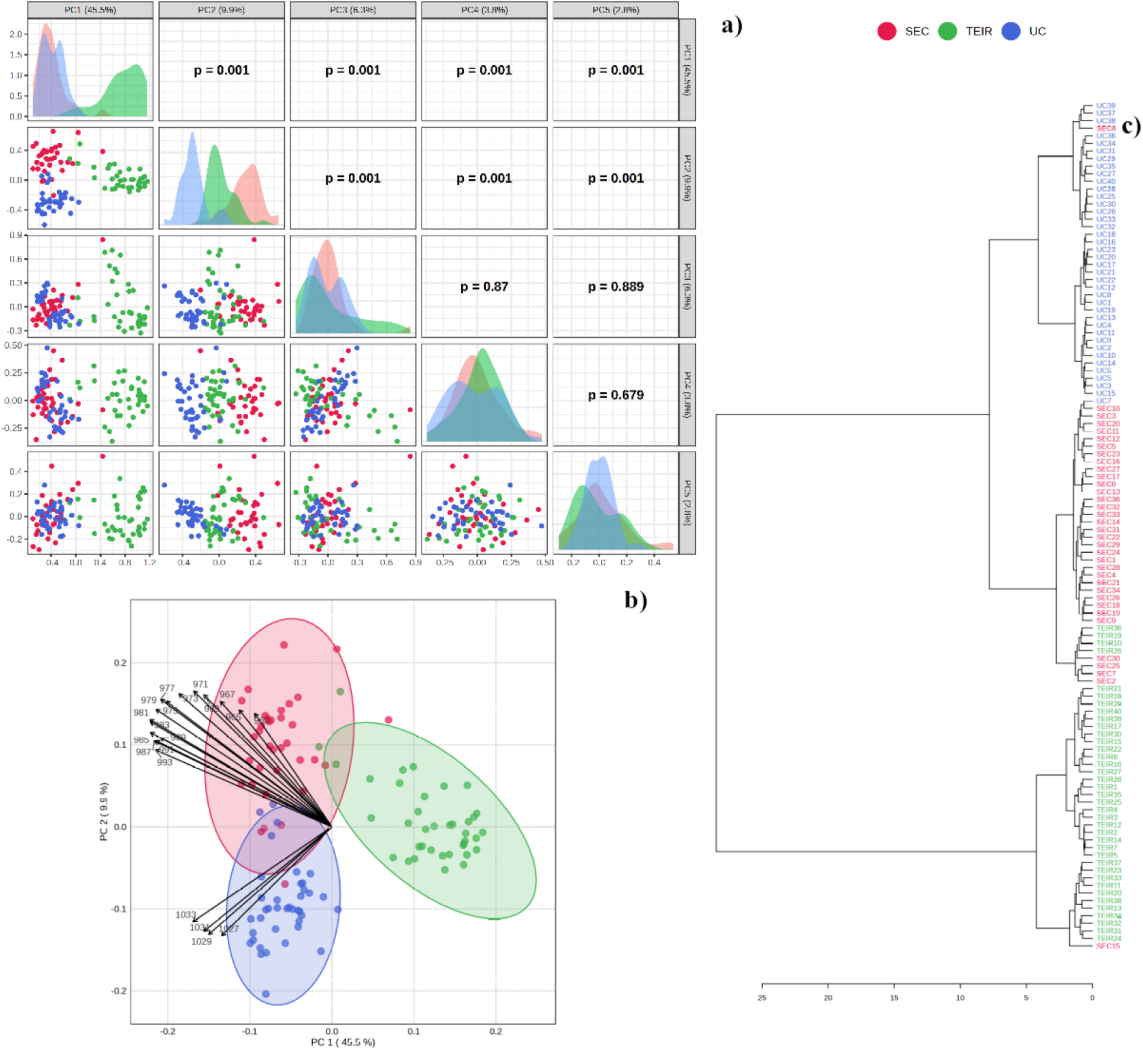
Principal component analysis (PCA) summary for size-exclusion chromatography (SEC), total exosome isolation reagent (TEIR), and ultracentrifugation (UC) methods of EV isolation, showing principal component pair combinations (a), a biplot for PC1 and PC2 showing the variance ellipses (b), and a dendrogram of spectra (c).

A similar trend was revealed by the supervised PLS-DA method (Figure 5). The discrimination among groups was also confirmed, as can be observed in the pairs score plot (Figure 5a), with the discriminative bands being basically the same (Figure 5b). The accuracy of the discrimination was 93% (Figure 5c). Thus, we can conclude that the EV preparation method decisively impacts their biochemical composition, and it is crucial to identify and isolate these key exogenous changes.

**Figure 5:**
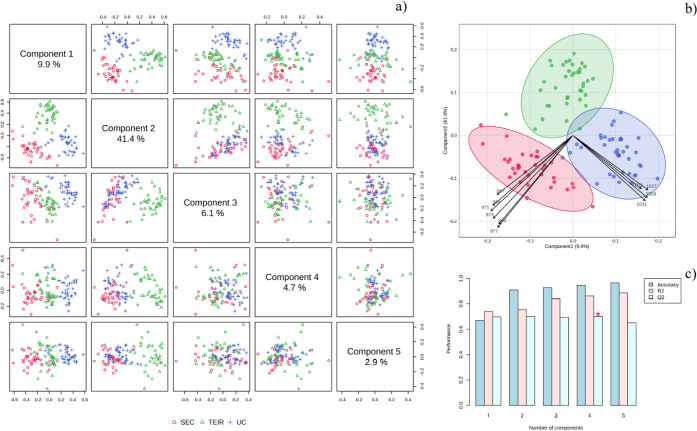
Partial least squares discriminant analysis (PLS-DA) summary for size-exclusion chromatography (SEC), total exosome isolation reagent (TEIR), and ultracentrifugation (UC) methods of EV isolation, showing principal component pair combinations (a), a biplot for PC1 and PC2 showing the variance ellipses (b), and performance parameters (c).

### Deconvolution of Albumin Contribution

At this point, it is important to identify spectral similarities and dissimilarities of groups, aiming to determine the characteristic EV spectrum. Then, we computed the average spectra of each group compared to the main source of non-EV cellular components bound to the EV surface, the albumin. We fitted the albumin spectrum to a sum of pseudo-Voigt line shapes and adjusted each albumin contribution to the SEC, TEIR, and UC spectra (Figure 6). Then, we deconvoluted the raw data to remove the bands related to albumin and observe an estimate of the pure EV spectrum for each case.

**Figure 6:**
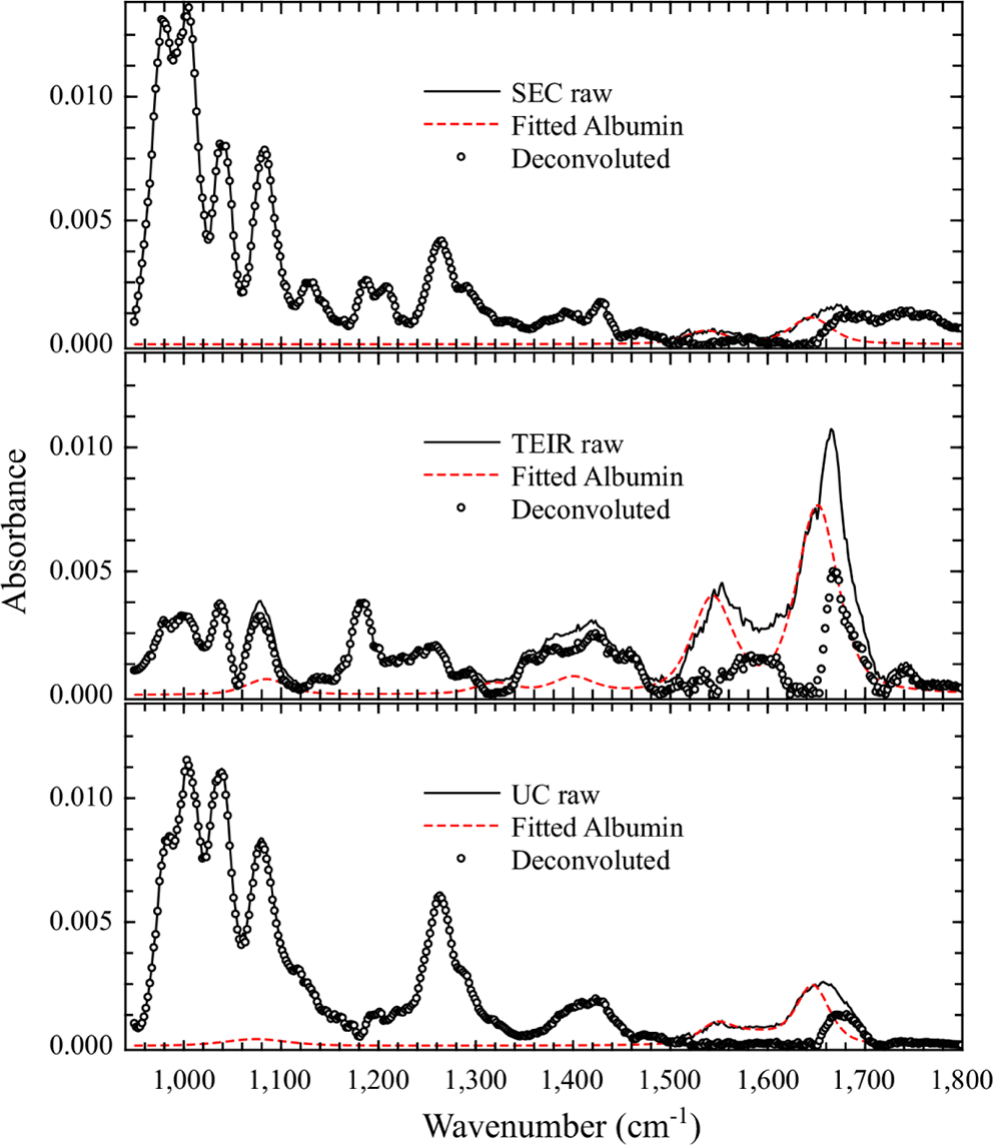
Processing of raw SEC, TEIR, and UC spectra to extract the albumin contribution. Solid lines represent the raw data, dashed red lines represent the sum of pseudo-Voigt lineshapes obtained from deconvolution of albumin spectra, and open circles represent the deconvoluted EV spectra in each case.

The albumin-deconvoluted EV average spectra are shown in Figure 7. At first glance, attention is drawn to the significant differences among spectra, which indicates that every processing method had a noticeable structural impact on the EV membrane.

**Figure 7:**
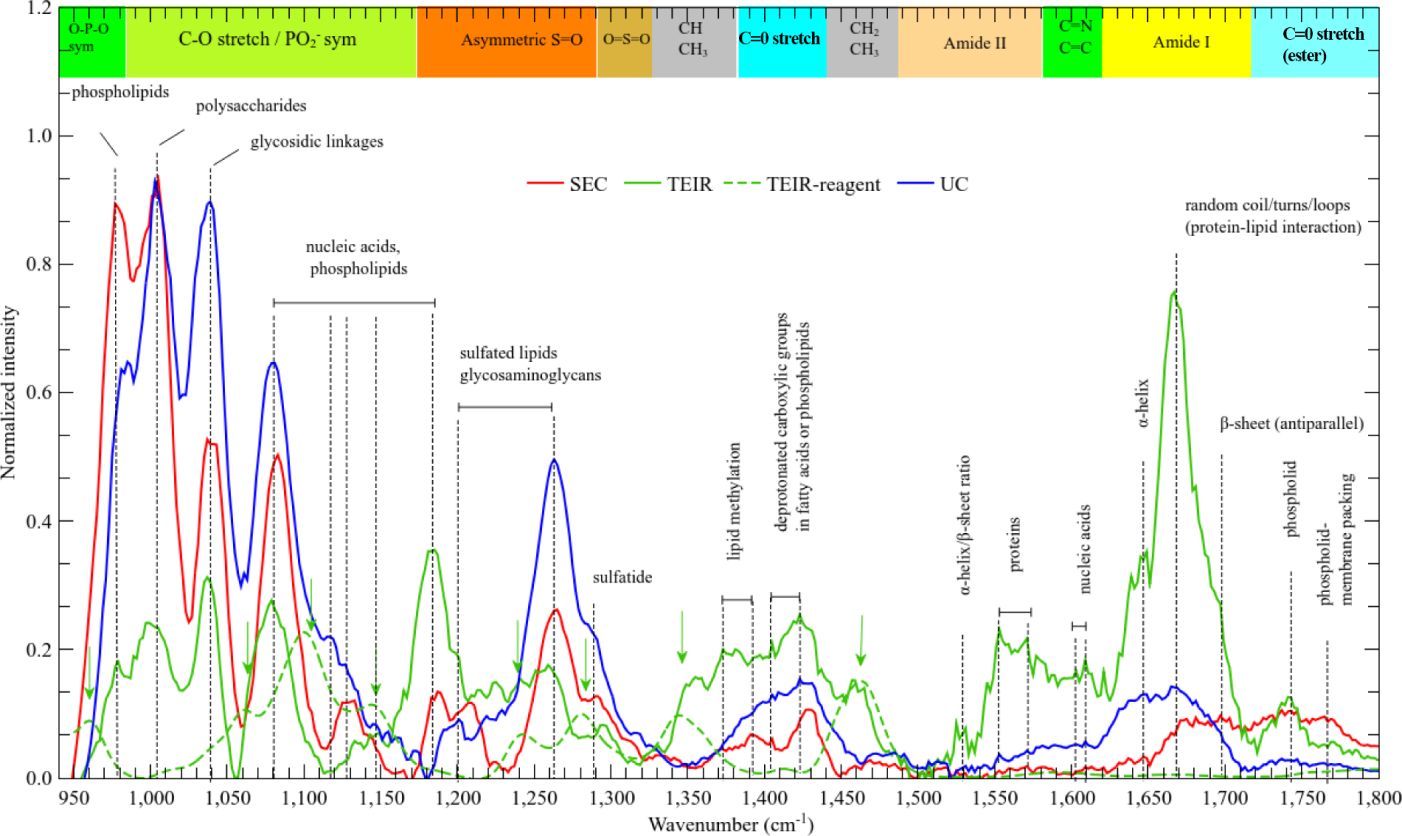
Average spectra of extracellular vesicles isolated under SEC, TEIR, and UC methods, with the corresponding band assignments. All these spectra were albumin-deconvoluted. The TEIR reagent spectrum is also shown.

The SEC processing strongly depletes the amount of biomolecules whose bands fall in the region above 1150 cm^–1^ which includes the α-helix and fatty acids content of EVs. Interestingly, the β-sheet and random coil/turn/loop conformational content of proteins, nucleic acids, phospholipids, carbohydrates, and glycoproteins content is almost preserved for SEC. On the other hand, the TEIR method is able to sustain the diverse fractions of conformational content of proteins, at the cost of damaging nucleic acids, phospholipids, carbohydrates, and glycoproteins content. Another aspect to be considered in TEIR is the remnant content of TEIR-reagent bond to the membrane. When comparing EV TEIR spectra to TEIR-reagent one, there are clearly discernible key bands (indicated by vertical arrows) still present. Still, the UC processing was able to preserve the largest amount of biomolecules present in EVs. However, structural damage on membranes was observed, e.g., smearing out of phospholipids related to membrane packing bands (1720–1800 cm^–1^ region) and the overall protein bands decreasing.

## Conclusions

The FTIR results largely corroborate the schematic spectrum proposed by the literature on infrared signatures from studies dedicated to characterizing extracellular vesicles from blood.^3,35,36^ However, the spectra also overlapped with albumin spectra, except for bands associated with triglycerides, membrane-bound oligosaccharides, lipids, and nucleic acid signatures. Nevertheless, as observed in topography maps from AFM-IR, the SEC EVs samples presented better topography of single vesicles and greater spectral homogeneity, even considering the complexity of the plasma sample. Additionally, the characteristic signature of albumin related to Amide II was not present in AFM-IR nanospectroscopy SEC results, probably associated with a decrease in the formation of a protein EV-corona, as previously described,^12^ and confirmed by our results.

On the other hand, it is important to address that the decrease of Amide I and the presence of Amide II peaks have been previously studied as a potential confirmation of blood contaminant depletion in the characterization of circulating EVs by IR spectroscopy associated with plasmonics,^35^ with Amide II related to the lesser contribution of these contaminants. In this way, further investigation regarding the Amide I and Amide II peaks in EVs according to their isolation method, as well as according to their biofluid origin, is needed (Tables 1,2).

**Table 1:** Mid-Infrared (IR) Assignments of the EV Samples Isolated by TEIR, UC, and SEC^3,16,17^

Wavenumber (cm^–1^)						
TEIR		UC		SEC		
FTIR	AFM-IR	FTIR	AFM-IR	FTIR	AFM-IR	Assignment
	1767	1770			1767	C=O stretching-phospholipid-membrane packing
1745	1743	1745		1745	1743	C=O stretching-phospholipid
1692	1700	1695	1700	1693	1700	Amide I - β-sheet (antiparallel)
1667	1680	1660–1639	1665	1660–1639	1676	Amide I random coil/turn/loop (protein–lipid interaction)
1642	1645	1632	1645	1636		Amide I α-helix
	1600–1615		1600–1615		1600–1615	C=N, C=C, nucleic acids
1542	1550–1575	1542	1550–1575	1542	1550–1575	Amide II – proteins
1520	1530	1520		1520		Amide II α-helix/β-sheet ratio
1397	1400–1430	1397	1400–1430	1397	1400–1430	deprotonated carboxylic groups in fatty acids or phospholipids
1373–1395	1373–1395	1373–1395	1373–1395	1373–1395	1373–1395	CH/CH_3_ – lipid methylation
1281	1295	1280	1289	1285	1292	Asymmetric S=O, sulfated lipids glycosaminoglycans
1251	1257	1251	1277	1251	1260	
1205	1204	1200	1200	1200	1200	
			1168			
1144	1123	1122	1130	1119	1129	C–O stretch/PO_2_^–^ of phospholipids, nucleic acids
			1116			C–O stretch/PO_2_^–^ of phospholipids, nucleic acids
1100	1078	1100	1080	1100	1084	υPO_2_^–^; phosphate vibration; symmetric phosphate [PO_2_^–^ (sym)] stretching; collagen and phosphodiester groups of nucleic acids
1032	1035	1027	1038	1030	1038	C–O stretch/PO_2_^–^ of glycosidic linkages
989	978	989	982	989	977	O–P–O symmetric stretching of phospholipids

**Table 2:** Key Negative Biochemical Impacts of Each EV Preparation Method

Method	Biochemical negative impact
SEC	α-helix and fatty acids content depletion
TEIR	TEIR reagent contamination
	Nucleic acids, phospholipids, carbohydrates, glycoproteins content depletion
UC	Phophospholipids, especially membrane phospholipids depletion

## Supplementary Material



## Notes

Regarding the micro-FTIR average spectra of all methods used and undiluted samples, AFM-IR topography, and spectral analysis are detailed in the Supporting Information (PDF)

## Abbreviations

AFM-IR: atomic force microscopy–infrared nanospectroscopy
EVs: extracellular vesicles
mi-IR spectroscopy: mid-infrared spectroscopy
FTIR: Fourier transform infrared spectroscopy
TEIR: Total Exosome Isolation Reagent
PBS: phosphate-buffered saline
SEC: size exclusion chromatography
NTA: nanoparticle-tracking analysis
DLS: dynamic light scattering
MISEV: minimal information for studies of extracellular vesicles
IR: infrared
s-SNOM: scanning near-field optical microscopy
PTIR: photothermal-induced resonance
PES: poly­(ether sulfone)
UC: ultracentrifugation
EDTA: ethylenediaminetetraacetic acid
DNA: DNA
RNA: ribonucleic acid
