# Supplementary material for: The Comparison between Different Extracellular Vesicle Isolation Methods by AFM-IR Nanospectroscopy
Source: Anal Chem. 2026 Mar 10;98(11):8289–99. doi: 10.1021/acs.analchem.5c07289 (PMC13019423; doi:10.1021/acs.analchem.5c07289)
Supplement: Supplementary file 1 [file ac5c07289_si_001.pdf]

# Supplementary Material

# The comparison between different extracellular vesicle isolation methods by AFM-IR nanospectroscopy

*Jéssica Verônica da Silva[1], Otávio Berenguel[2], Raquel Silva Neres-Santos[1], Herculano da Silva Martinho[1]* and Marcela Sorelli Carneiro-Ramos[1]*.*

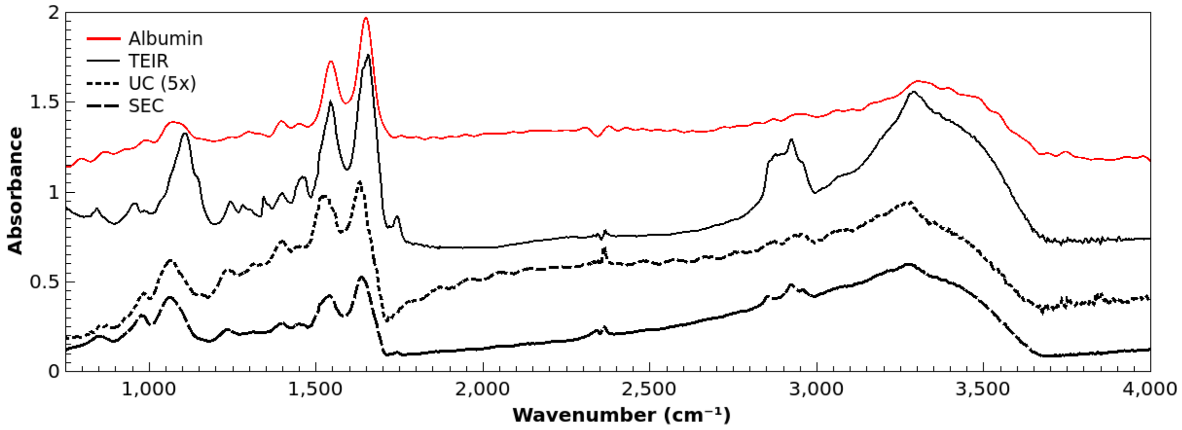


**Figure S1**. Average micro-FTIR spectra from 950 - 4000 $cm^{-1}$ for the albumin, Total Exosome Isolation Reagent (TEIR), ultracentrifugation (UC), and size exclusion chromatography (SEC) isolated EVs (Varian 610/640 FTIR).

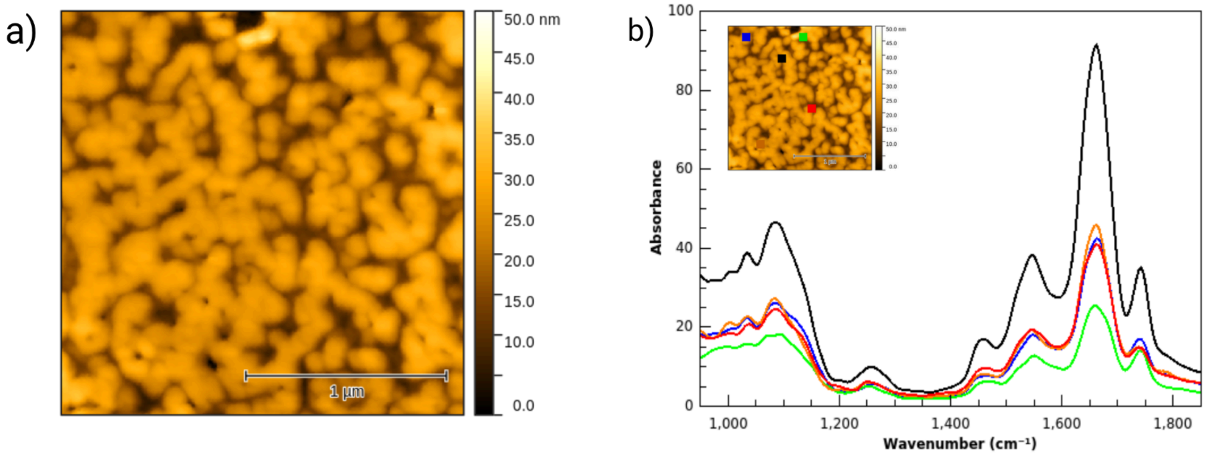


**Figure S2.** AFM-IR analysis of the TEIR EVs (1:1) sample. (a) Topographic image (2 x 2 µm²) on the left with the points corresponding to the (b) IR spectra on the right. As observed, in the undiluted sample, a topography and spectral collection of individual EVs is not effective regardless of the sample's concentration.

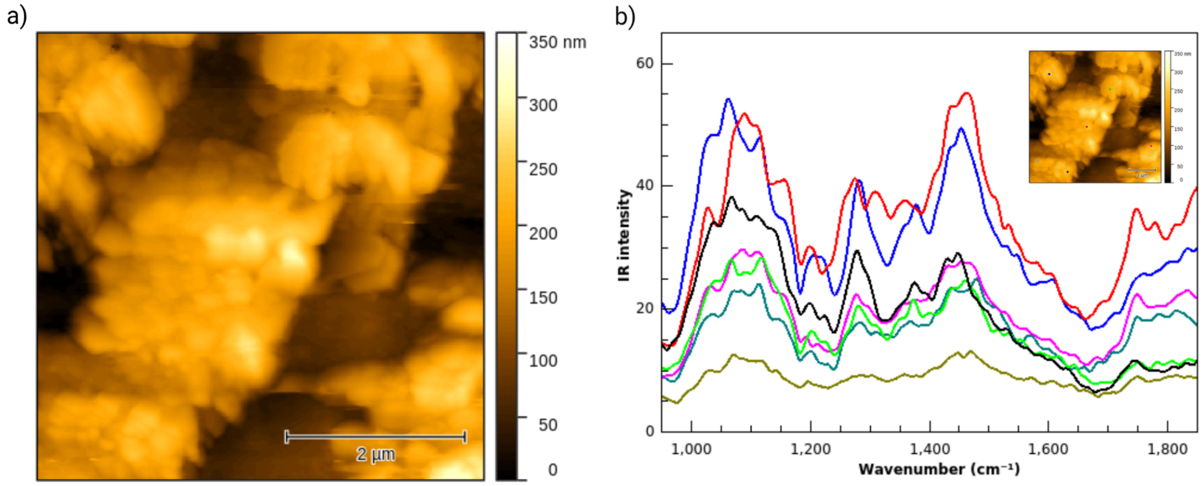


**Figure S3.** AFM-IR analysis performed in the albumin at different points: a) topographic image (5 x 5 µm²) on the left with the points corresponding to the b) IR spectra on the right.

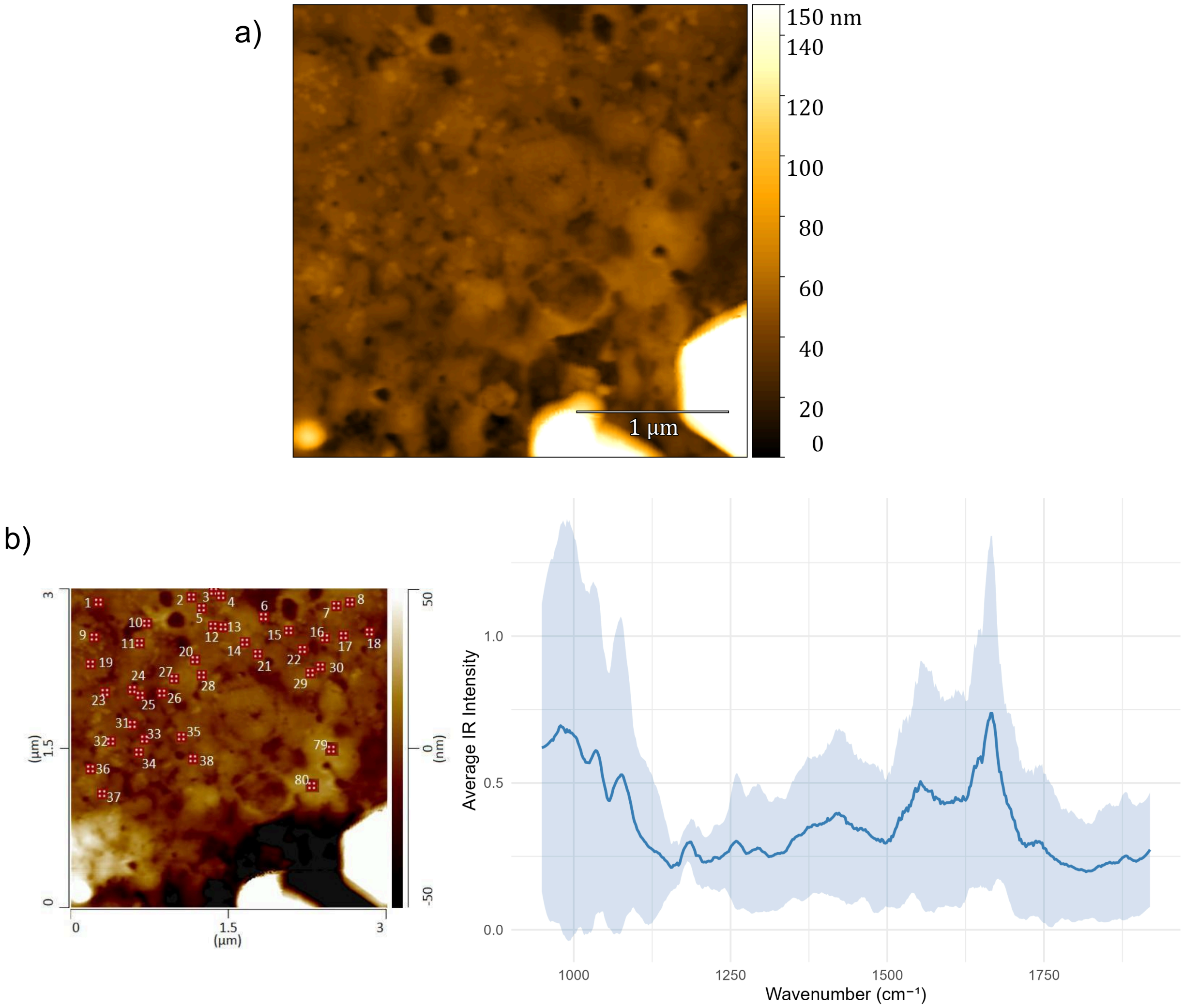


**Figure S4.** AFM-IR analysis of the TEIR EVs (1:10) sample. (a) Topographic image (3 x 3 µm²) on the upper part of the figure, and (b) the corresponding average IR spectra on QCL.

a)

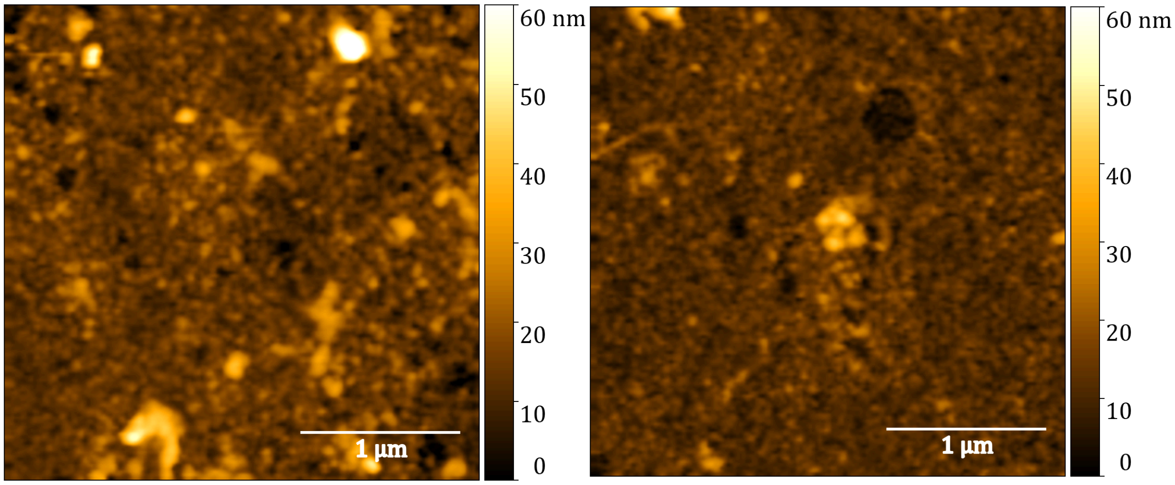


b)

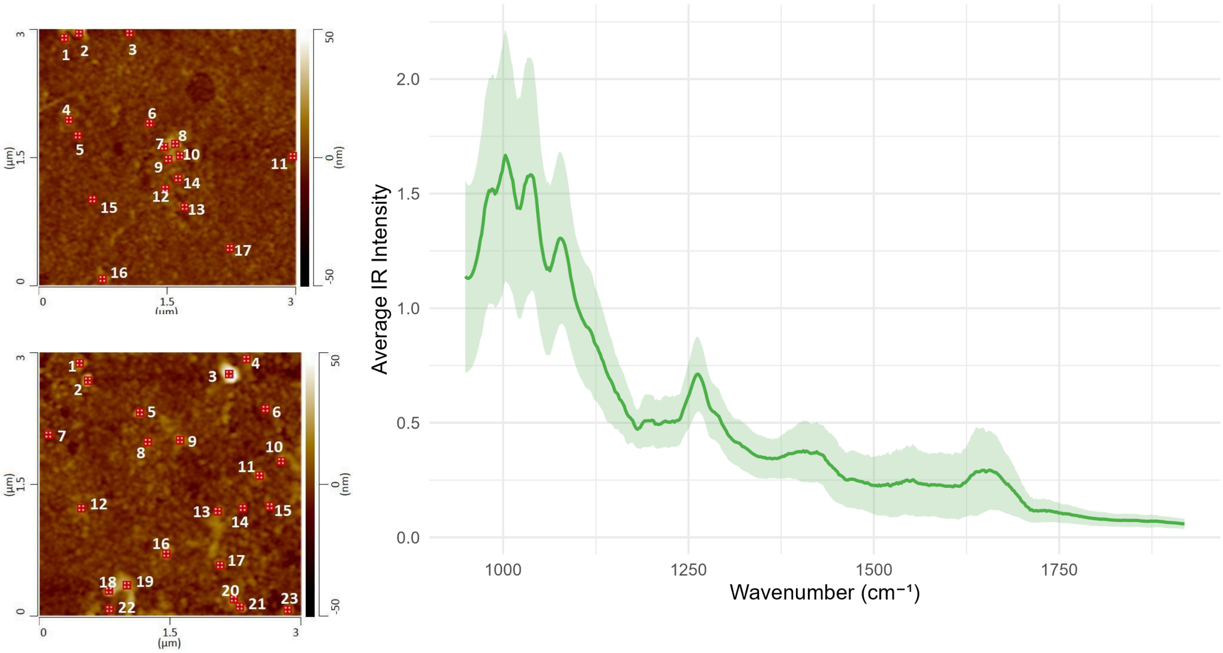


**Figure S5.** AFM-IR analysis of the UC EVs (1:10) sample. (a) Topographic images (3 x 3 µm²) in two different regions on the upper part of the figure, and (b) the corresponding average IR spectra on QCL.

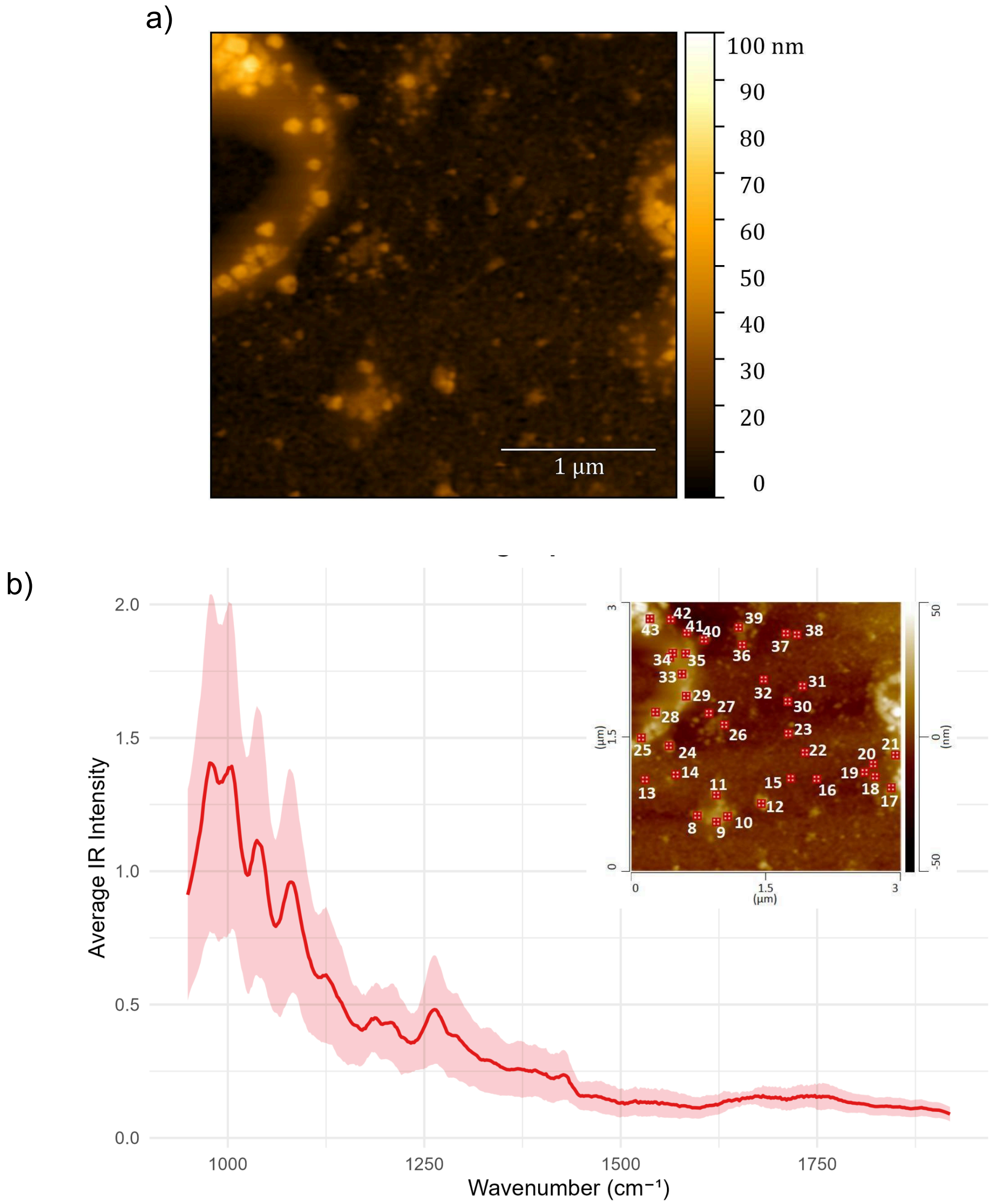


**Figure S6.** AFM-IR analysis of the SEC EVs (1:10) sample. (a) Topographic image (3 x 3 µm²) on the upper part of the figure, and (b) the corresponding average IR spectra on QCL.
